# Supplementary material for: Dynamics-dependent density distribution in active suspensions
Source: Nat Commun. 2019 May 24;10:2321. doi: 10.1038/s41467-019-10283-0 (PMC6534614; doi:10.1038/s41467-019-10283-0)
Supplement: Supplementary file 1 — Supplementary Information [file 41467_2019_10283_MOESM1_ESM.pdf]

Supplementary information for 'Dynamics dependent density distribution in active suspensions'

Jochen Arlt, Vincent A. Martinez, Angela Dawson, Teuta Pilizota and Wilson C. K. Poon

April 19, 2019

Supplementary Figures

1 Probability distributions of $\bar{v}$, β, ρ/ρ_0, and $\rho^s v/\rho_0$ as a function of tile size . . . 2
2 Spatial analysis of a uniformly illuminated sample for a range of tile sizes . . . 3
3 Corresponding full spatial maps for a tile size of 64 pixels . . . 4
4 sDDM maps for an intensity step projected onto a sample of AD10 at OD=1 . . . 5
5 Time evolution of y-averaged values vs. x position for AD10 (OD=1) sample . . . 6
6 Spatial maps characterising a dense suspension of DM1 (OD = 8) . . . 7
7 Time evolution of y-averaged values vs. x position for DM1 (OD = 8) sample . . . 8
8 Intensity-dependent non-motile fraction . . . 9
9 Time evolution of y-averaged values vs. x position for sample with $\beta(I)$ . . . 10
10 Spatial maps for a sample of light–powered AD4 WT swimmers (OD = 1) . . . 11
11 Time evolution of y-averaged values vs. x position for AD4 (OD = 1) sample . . . 11

Supplementary Tables

1 Light controlled *E. coli* strains used in this work . . . 12

Supplemenary Note

Supplementary Note 1 Spatially-resolved DDM **12**

Supplementary Note 2 Analytical expressions for simple 1D-speed profiles **12**

Supplementary Note 3 Intensity dependent non-motile fraction **13**

Supplementary Note 4 Run-and-tumble strain AD4 **13**

Supplementary Figures

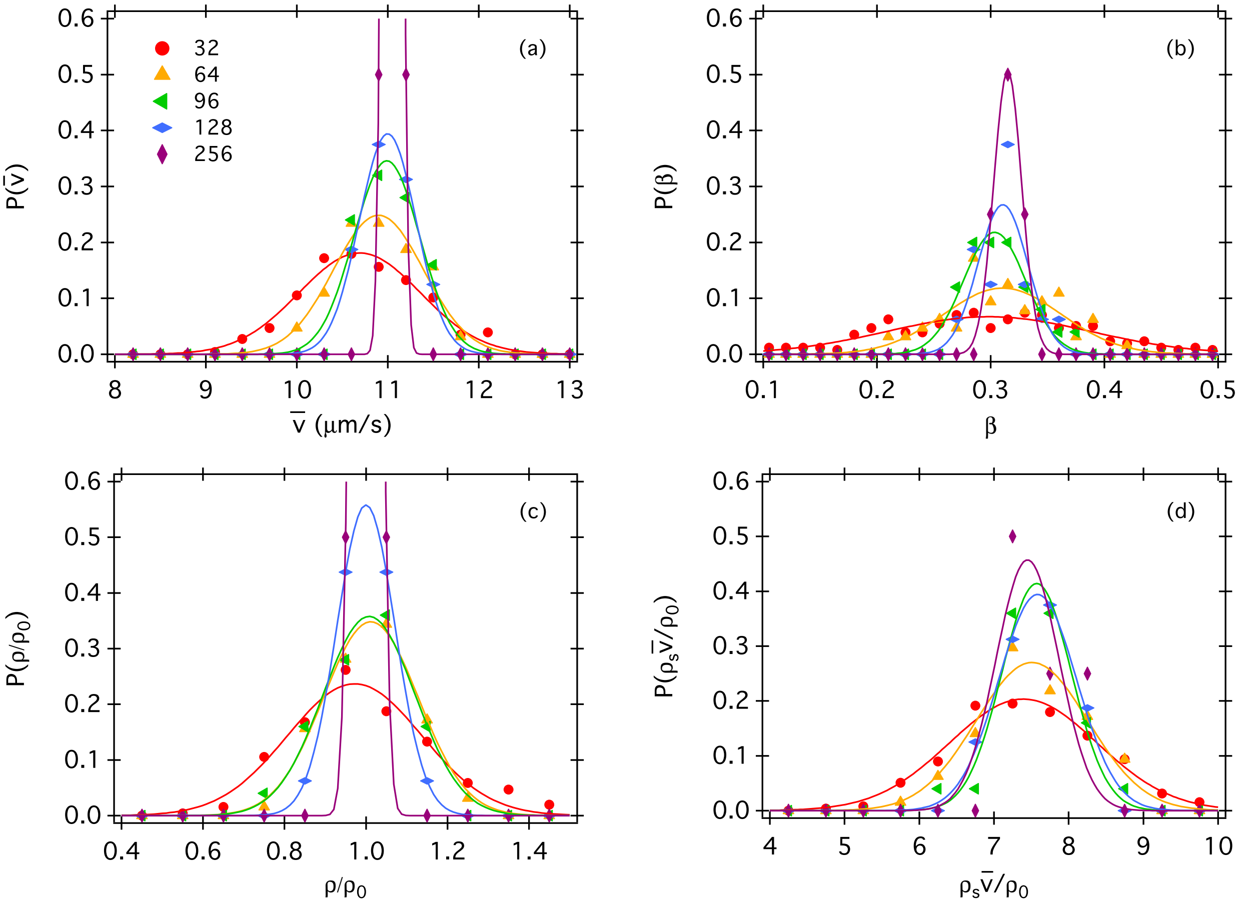

Supplementary Figure 1: Probability distributions of (a) mean swimming speed $\bar{v}$, (b) non-motile fraction β, (c) relative cell density ρ/ρ_0, and (d) $\rho^s v/\rho_0$ as a function of tile size (in pixels, see legend) for a uniform sample of *E. coli* DM1 at OD=1. Here ρ_0 corresponds to the average over (x, y), i.e. $\rho_0 = \langle\rho(x, y)\rangle_{x,y}$. Lines are Gaussian fits to the data.

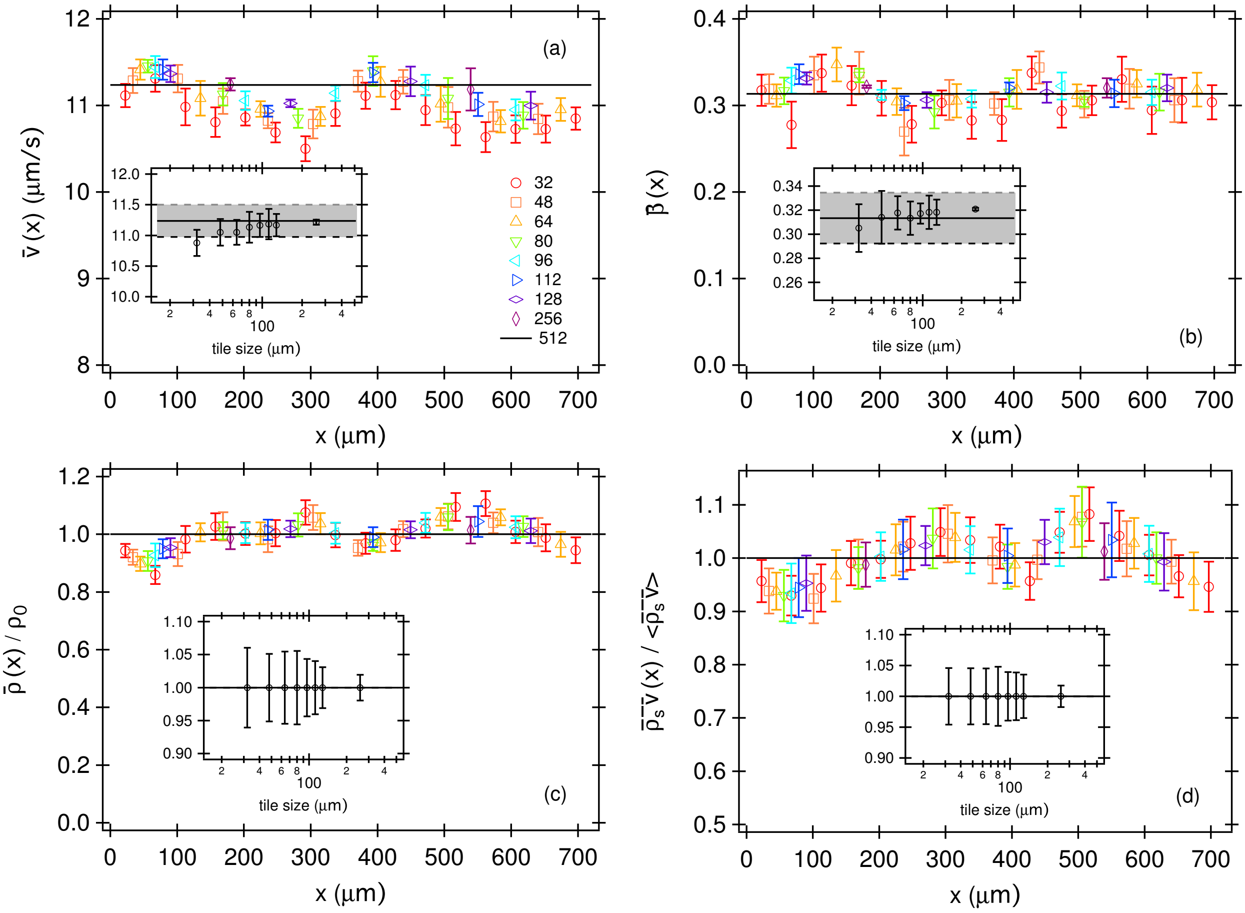

Supplementary Figure 2: Spatial analysis of a uniformly illuminated sample for a range of tile sizes (in pixels, see legend). a) mean swimming speed $\bar{v}$, b) non-motile fraction β, c) relative density ρ/ρ_0 and d) $\rho^s\bar{v}/\langle\rho^s\bar{v}\rangle$ averaged over all tiles along the y-axis vs position x (in microns, 1 pixel = 1.4 µm). $\langle\rho^s\bar{v}\rangle$ represents the average over (x, y). Insets show corresponding overall averages over x of these parameters as a function of tile size (in microns). Black lines and grey area corresponds to the mean value and its error obtained from the whole field of view (i.e. tile size=512 pixels). Error bars in main plots are error of the mean from averaging over the y-axis. Error bars in insets are standard deviations of the mean by averaging over x-axis.

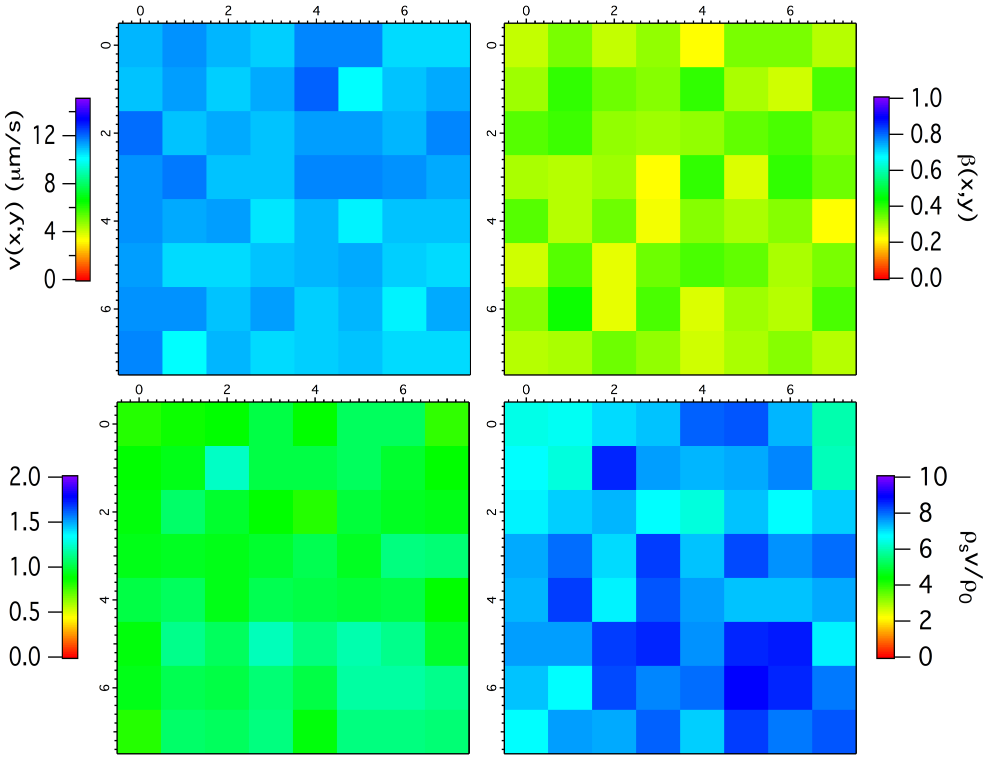

Supplementary Figure 3: Corresponding full spatial maps for a tile size of 64 pixels (as used throughout the manuscript) for the plots shown in Supplementary Figure 2.

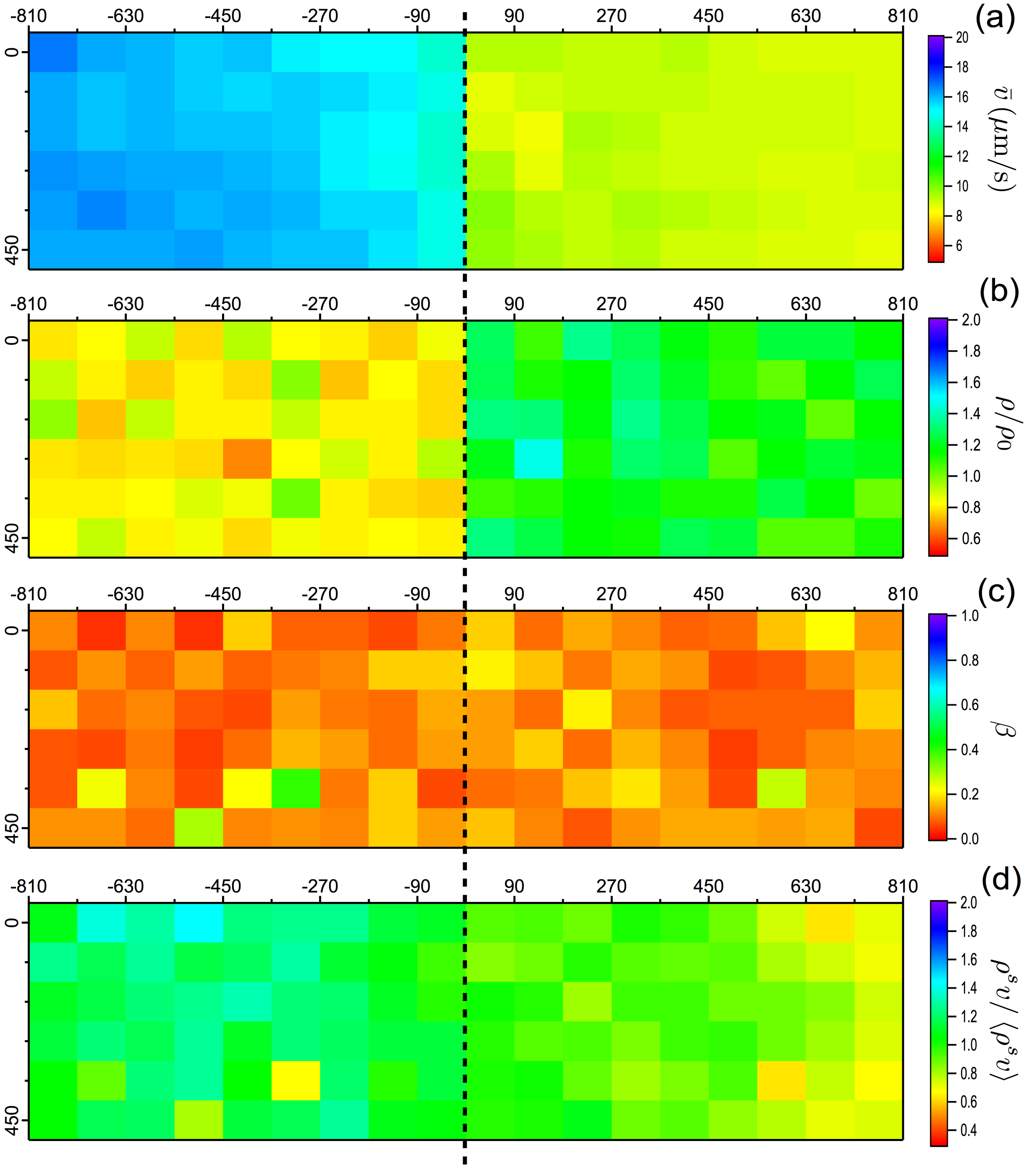

Supplementary Figure 4: sDDM maps for an intensity step projected onto a sample of AD10 at OD=1 for $\approx 29\,\text{min}$. (a) swimming speed $\bar{v}(x, y)$, (b) total density $\rho(x, y)$ relative to the density of the uniform sample $\rho_0 = \langle\rho_0\,(x, y)\rangle_{x,y}$ just before switching on the step pattern, (c) non-motile fraction $\beta(x, y)$ and (d) $\rho^s v(x, y)$ normalised by its average over the whole map $\langle\rho_s v\,(x, y)\rangle_{x,y}$. Dotted line is boundary of the two halves pattern. Spatial axis in µm.

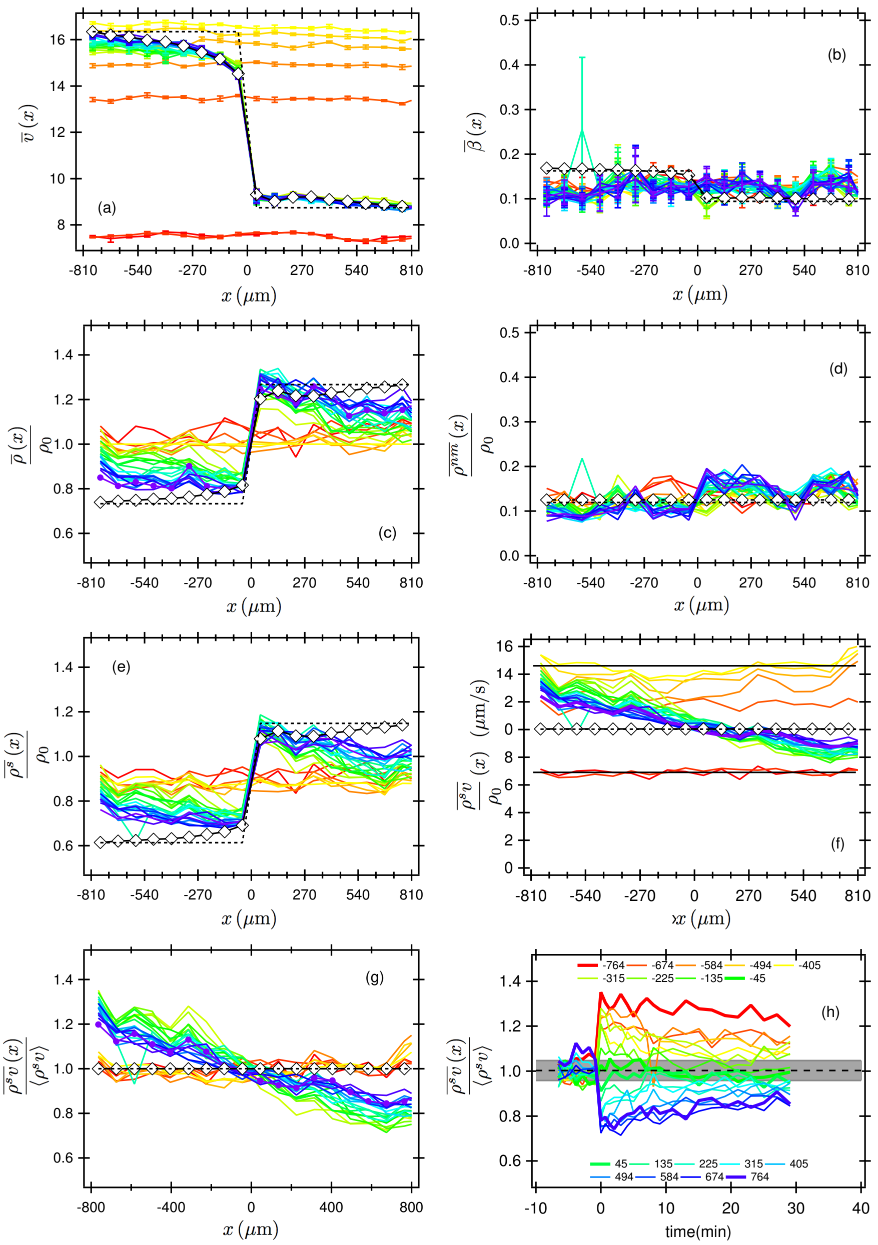

Supplementary Figure 5: Time evolution of y-averaged values vs. x position for: (a) $\bar{v}(x)$, (b) $\bar{\beta}(x)$, (c) $\bar{\rho}(x)/\rho_0$, (d) $\bar{\rho}^{\,\mathrm{nm}}(x)/\rho_0 = \bar{\beta}(x)\bar{\rho}(x)\rho_0$, (e) $\bar{\rho}^{s}(x)\rho_0 = (1-\bar{\beta}(x))\bar{\rho}(x)\rho_0$, (f) $\bar{\rho}^{s}\bar{v}$, where thick lines are the corresponding values for the uniform case for high and low intensities, and (g) normalised values of (f). For (a-g) colour encodes time on a rainbow scale. red to yellow: uniform illumination switched from low intensity to high intensity just after the red data set. Two halves pattern switched on just after yellow. Dotted black lines are prediction using two speeds only (values used are represented in the (a)). Black diamonds are theoretical prediction considering $v(x)$ of the last time-point of the two halves pattern (purple line in all plots but the last bottom right). (h) time evolution as plotted for the individual x positions as indicated in legend. The halves pattern is applied at $t = 0$. At $t < 0$, uniform conditions allows determination of the noise which corresponds to the grey area.

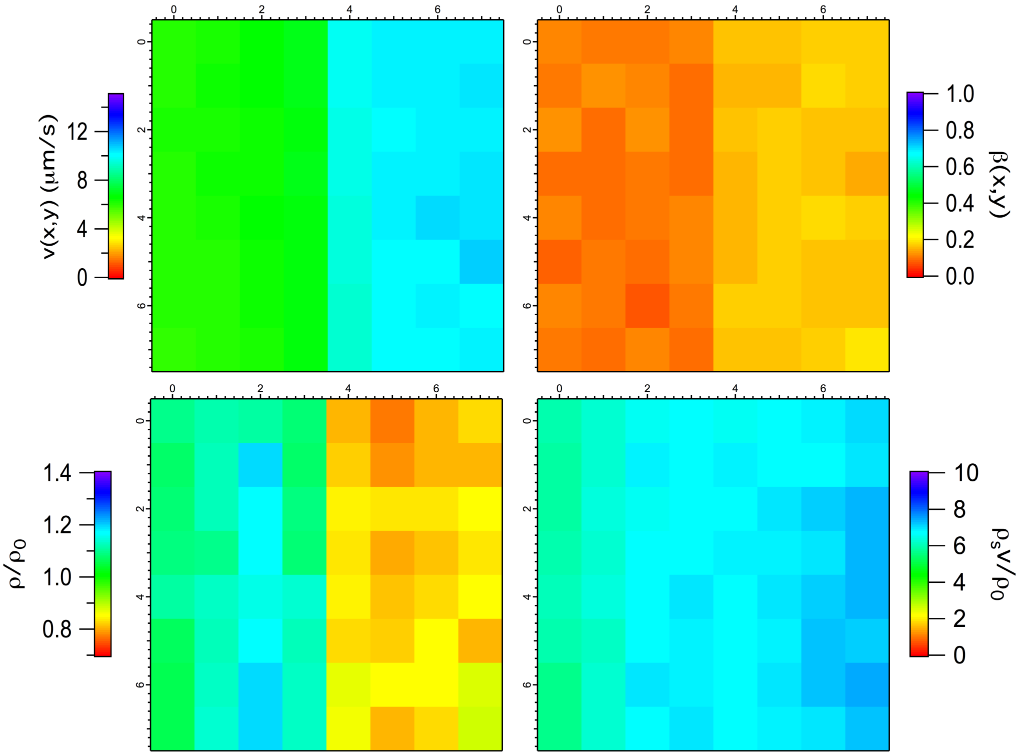

Supplementary Figure 6: Spatial maps characterising a dense suspension of DM1 (OD = 8) near the boundary between a low (left) and high (right) light intensity region after $t \approx 20$ min of illumination: (a) mean speed $\bar{v}$, (b) non-motile fraction β, (c) relative cell density ρ/ρ_0, and calculated from those (d) $\rho_s v/\rho_0 = \rho(1-\beta)v/\rho_0$. Each tile corresponds to 64×64 pixel^2, or $90 \times 90\,\mu\text{m}^2$.

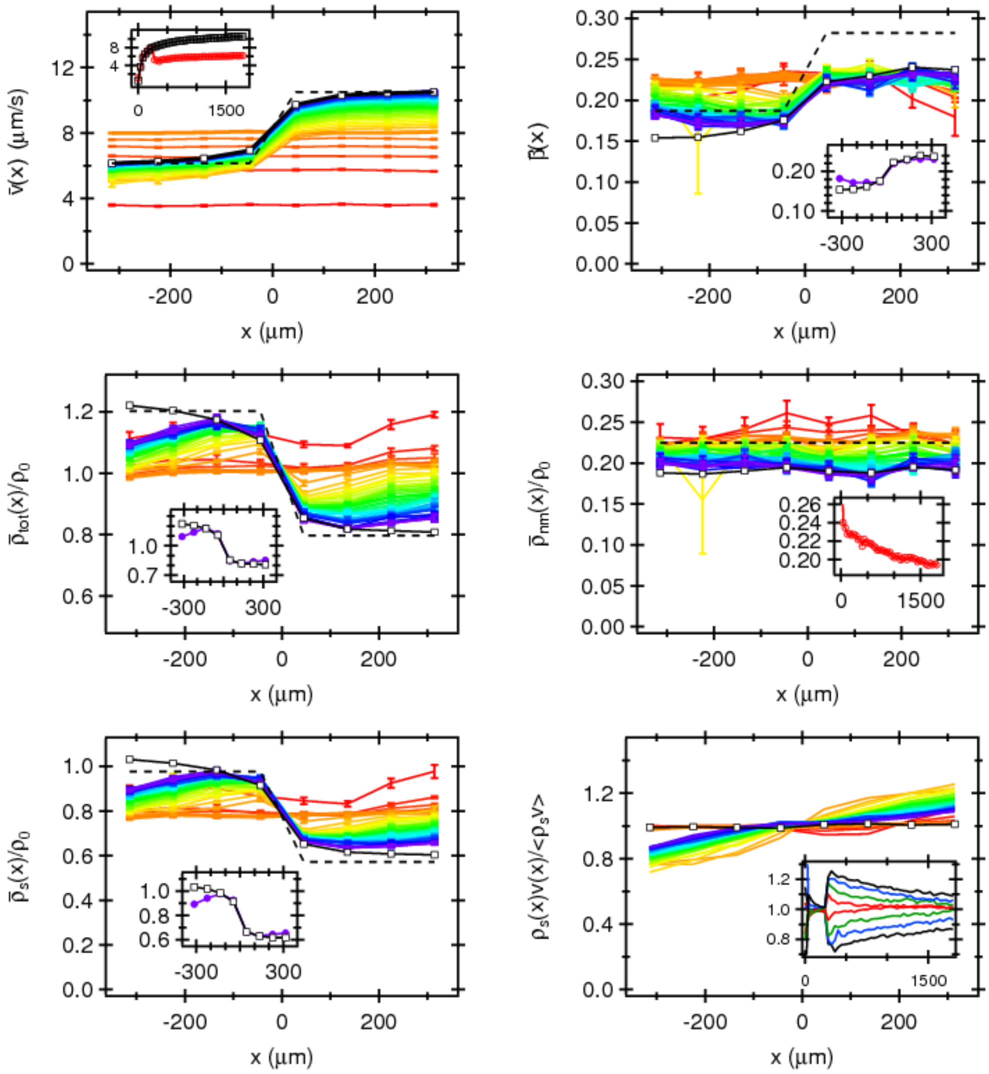

Supplementary Figure 7: Time-dependency of the y-averaged values along x for: (a) $\bar{v}(x)$, (b) $\bar{\beta}(x)$, (c) $\bar{\rho}(x)/\rho_0$, (d) $\bar{\rho}^{\mathrm{nm}}(x)/\rho_0 = \bar{\beta}(x)\bar{\rho}(x)\rho_0$, (e)$\bar{\rho}^s(x)\rho_0 = (1-\bar{\beta}(x))\bar{\rho}(x)\rho_0$ and (f) the normalised $\overline{\rho^s v}$. Inset in (a): speed versus time (in seconds) for the two extreme tiles. Initially the sample was illuminated uniformly for $\approx 4\,\mathrm{min}$ and both speeds are equal. Then the stepped light pattern is switched on for $\approx 16\,\mathrm{min}$. Thick orange lines mark the uniform state just before applying the pattern. Insets in (b, c, e) are zoomed in for better comparison between experiments and theory. Inset in (d) is the mean value across the sample to highlight decrease in ρ^{nm}. Inset in (f) shows the time-dependency of the normalised $\overline{\rho^s v}$ for each tile (or x). All tiles but the two extremes (corresponding to a distance of 315μm from boundary) seem to converge to one. Dotted lines: theory from simple two binary speeds with β_0 measured from last uniform movie before switching on the stepped light pattern. Black square lines: theory from multiple sub-regions with β_0 obtained by using Eq. 4, and thus taking into account the loss of non-motile cells over time presumably correlated with reaching the motility saturation.

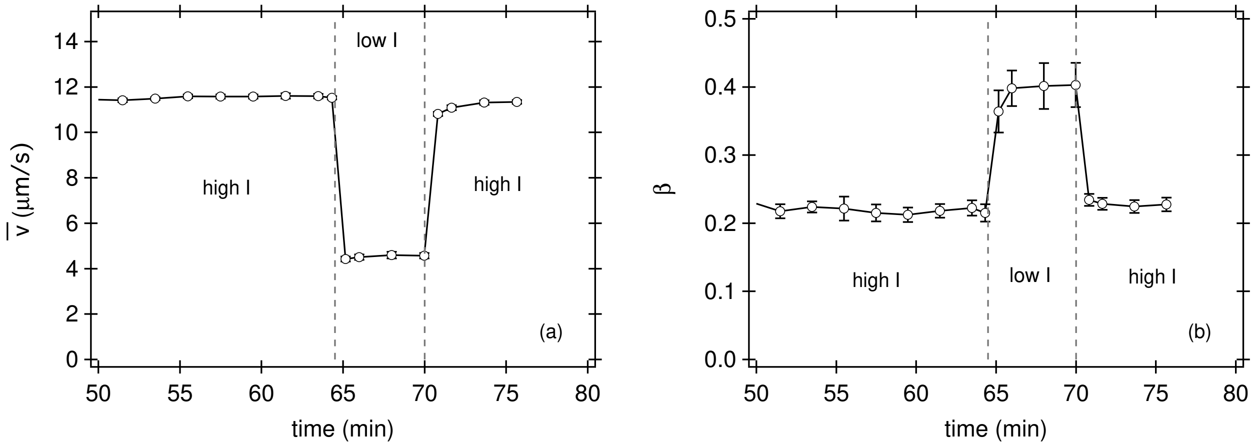

Supplementary Figure 8: Intensity-dependent non-motile fraction. (a) Mean swimming speed $\bar{v}$ and (b) non-motile fraction β for spatially uniform illumination as a function of time. Intensity is switched from high to low and low to high as indicated by dotted lines.

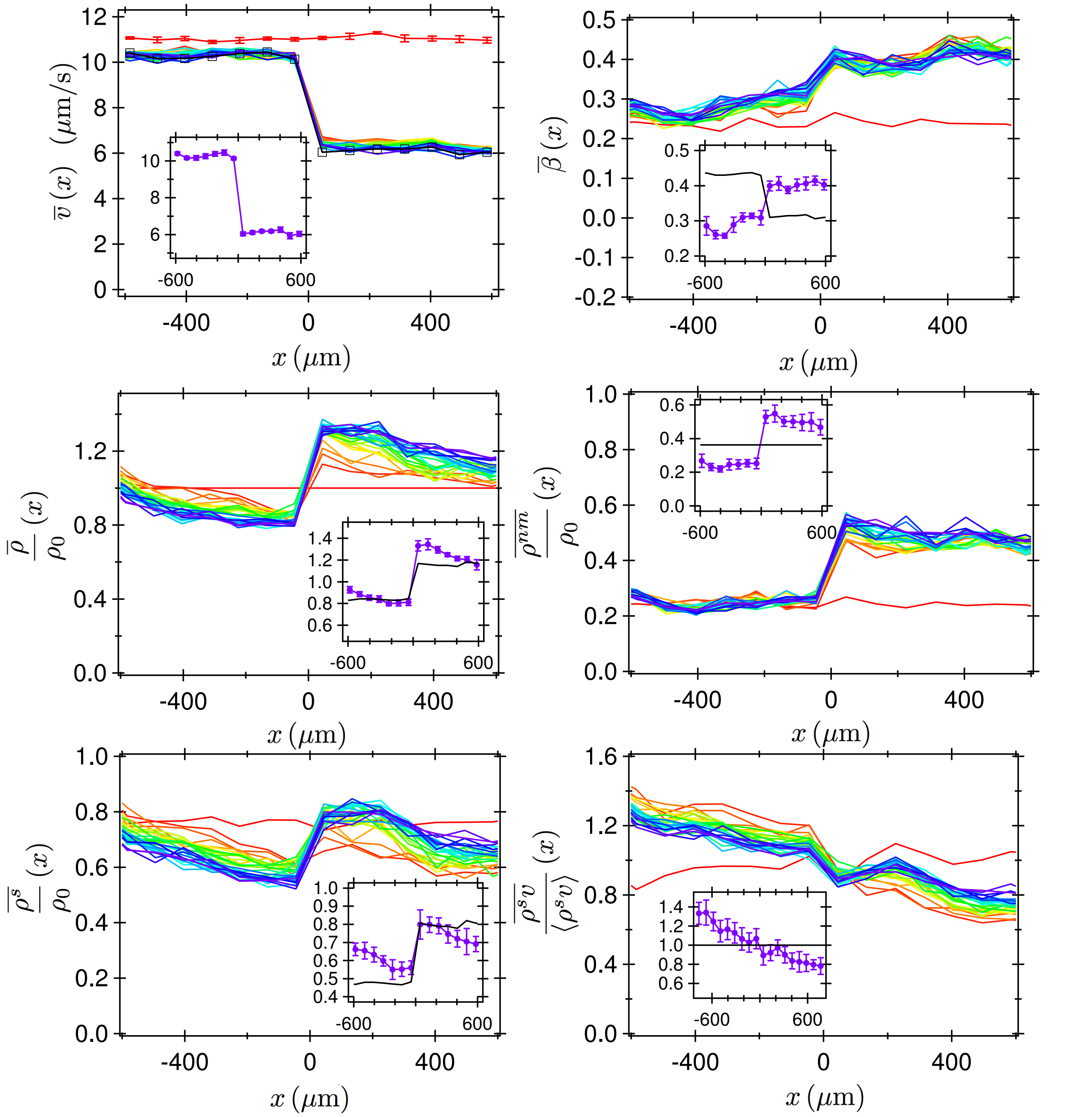

Supplementary Figure 9: Time evolution of profiles for a stepped light pattern experiment for which the non-motile fraction β displays significant dependency with the light intensity in uniform illumination condition. Insets show last experimental dataset ($t \approx 25\,\mathrm{min}$) together with the theoretical predictions (black line) assuming a constant non-motile density (eqs. (4), (3) & (2)).

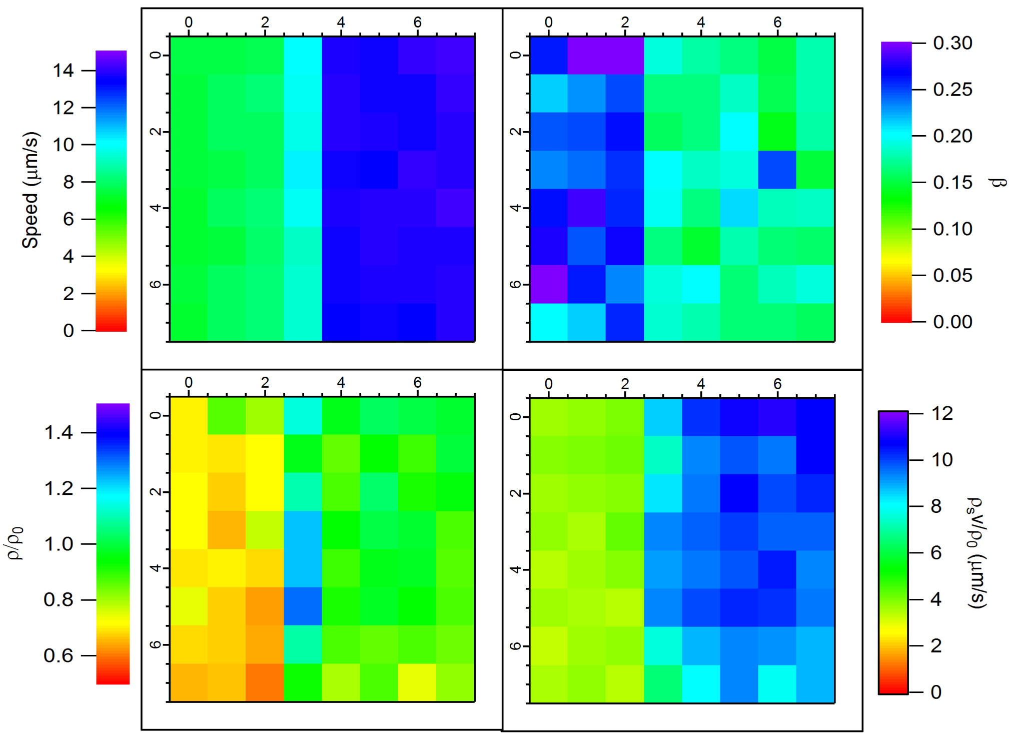

Supplementary Figure 10: Spatial maps for a sample of light–powered WT swimmers (strain AD4, OD = 1) after 17 min of illumination with a step pattern: mean speed $\bar{v}$, non-motile fraction β, relative total density ρ/ρ_0 and $\rho^s v/\rho_0$.

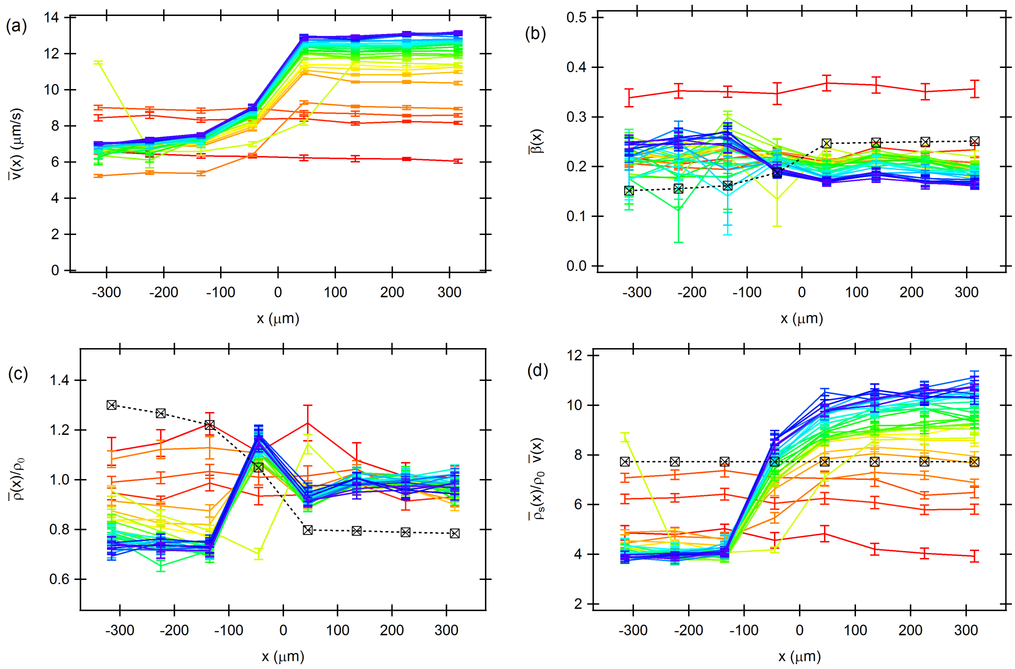

Supplementary Figure 11: Time series ($\Delta t = 40\,\mathrm{s}$) of profiles from initially uniformly illuminated sample (red) to switching on the stepped intensity pattern (orange) to final profile (purple) corresponding to the maps shown in Supplementary Figure 10. (a) mean speed $\bar{v}(x)$, (b) non-motile fraction $\bar{\beta}$, (c) relative total density $\bar{\rho}(x)/\rho_0$ and (d) $\overline{\rho_s v}(x)/\rho_0$. Black squares indicate the predictions based on eqs. (4), (3) & (2)) and the measured final mean speed profile $\bar{v}(x)$.

Supplementary Tables

Name	Motility genotype	Parent strain	Relevant genotype
DM1	smooth swimmer	RP437	DE3 $\Delta(unc)$ + $\Delta(CheY)$ + pET200/ PR-GFP
AD4	motility WT	AB1157	$\Delta(unc)$ + pBAD-HisC-PR
AD10	smooth swimmer	AB1157	$\Delta(unc)$ + $\Delta(CheY)$ + pBAD-HisC-PR

Supplementary Table 1: Light controlled *E. coli* strains used in this work. The two smooth swimming strains are underlined; they were used for the majority of results presented in the manuscript, e.g. the data summarised in fig. 3c.

Supplementary Note 1 Spatially-resolved DDM

In order to test the validity of sDDM on typical movies used in this work we first analysed spatially uniform samples by dividing them into tiles of progressively smaller sizes. Supplementary Figure 1 shows that the mean values of swimming speed $\bar{v}$, non-motile fraction β and relative density ρ/ρ_0 are independent of tile size within errors. From these measured values we can then estimate the (relative) swimmer flux $\rho^s \bar{v}/\rho_0$ by calculating $(1-\beta_i)\rho_i \bar{v}_i/\rho_0$ for each of the tiles, which again has a mean which is independent of tile size (Supplementary Figure 1d). The corresponding spatial profiles shown in Supplementary Figure 2 show that the swimming speed, non-motile fraction as well as relative density and swimmer flux are all spatially constant within the errors. For the data presented in the main manuscript we choose a tile size of 64 pixels square (see Supplementary Figure 3 for resulting maps of the uniform sample) to provide a compromise between spatial resolution and noise in the measured quantities. For some of the 1.6 mm wide data sets the relative density profiles needed a correction factor applied to account for lower DDM signal amplitudes near the edge of the field of view.

Supplementary Note 2 Analytical expressions for simple 1D-speed profiles

In practice, there is often a non-negligible fraction of non-motile cells (primarily due to the filtration process), thus adding additional parameters to the problem. From sDDM, we measure the total density ρ and the non-motile fraction β, allowing calculation of the non-motile and swimmers densities, $\rho^{nm} = \beta\rho$ and $\rho^s = (1-\beta)\rho$ respectively. In the present study, we apply 1D speed profiles that vary only in the x-direction. In this section, we derive expression for the non-motile fraction $\beta(x)$, swimmer $\rho^s(x)$, non-motile $\rho^{nm}(x)$, and total $\rho(x)$ densities based on three extra assumptions:

(i) For simplicity we assume that the complete sample is sub-divided into N equal volume elements, each of which has a prescribed speed v_i. All the parameters can thus be represented on an equidistant grid, directly corresponding to the spatial grid of sDDM.

(ii) We assume conservation of total particle number so that $\rho = \rho_0 = \frac{1}{N}\sum_i \rho_i$ with ρ_i the total cell density in sub-region i. Experimentally this seemingly trivial assumption is actually rather difficult to implement: if the sample is not illuminated completely but has dark regions these act as 'sinks', as swimmers completely stop swimming there and lead to a continuous drop of the active population [1]. We therefore took care to illuminate as much as the sample chamber as possible (i.e. illuminating a 7 mm diameter area much larger than the field of view recorded by our camera).

(iii) We assume a constant non-motile density across the field of view and experimental time window, i.e.

$$\rho_i^{nm} = \beta_i \rho_i = \beta_0 \rho_0 \tag{1}$$

with β_0 and ρ_0 the (x, y)-averaged values of the uniform distribution. This assumption is justified with two reasons. Firstly, the diffusion coefficient of non-motile cells are orders of magnitude smaller than the effective

diffusion coefficient of the swimmers, thus we expect the non-motile cell density evolves much more slowly away from this initially uniform spatial distribution. Secondly, previous studies have shown that the presence of swimmers enhanced the diffusion of non-motile cells [2, 3], so that one might expect different diffusion coefficients in the high and low speed regions, which could result in a net flux. However, it was found that this enhanced diffusion ΔD scales with the flux of the swimmers, i.e. $\Delta D \propto \rho^s v$ [2, 3], and thus a uniform distribution of non-motile cells across the field of view should be maintained provided $\rho^s(x)v(x) = \text{const}$.

Considering $\rho_i^s v_i$ =constant between neighbouring volumes and the three above assumptions lead to

$$\frac{\rho_i^s}{\rho_0} = \frac{1-\beta_0}{v_i \langle 1/v \rangle} \tag{2}$$

$$\frac{\rho_i}{\rho_0} = \frac{\rho_i^s}{\rho_0} + \beta_0 \tag{3}$$

$$\beta_i = \frac{\beta_0 \, v_i \langle 1/v \rangle}{1 + \beta_0 (v_i \langle 1/v \rangle - 1)}. \tag{4}$$

where $\left\langle \frac{1}{v} \right\rangle = \frac{1}{N} \Sigma_{i=1}^N \frac{1}{v_i}$ is the mean of the inverse speeds, ρ_0 the initial (total) density of the sample and β_0 the corresponding non-motile fraction.

For our single stepped pattern we only need two regions ($N = 2$), which leads to the simple eq. 10 presented in the main manuscript. But often the experimental speed profile shows a more gradual transition near the boundary. We therefore use the measured speeds v_i as input and calculate the expected swimmer density profile, such as shown in figures 2a & 3a of the main text and Supplementary Figures 5 & 7.

Supplementary Note 3 Intensity dependent non-motile fraction

In some experiments we detected a light intensity-dependence of the non-motile fraction under uniform illumination, i.e. $\beta_0(I)$. This was particularly evident when working at very low light intensities (i.e. when working at low swimming speeds). Supplementary Figure 8 shows an extreme case for which $\beta_0(\mathcal{I}_{\text{high}}) = 0.22$ and $\beta_0(\mathcal{I}_{\text{low}}) = 0.40$ at high and low intensity, respectively. This suggests that some of the cells which happily swim at high intensity will become non-motile at the lower intensity and only a reduced fraction of cells can maintain swimming at both intensities. This severely complicates the interpretation of stepped intensity pattern experiments as swimmers and non-motile cells can no longer be considered as two independent sub populations.

This is clearly highlighted by Supplementary Figure 9 which shows the time-dependent response of the same sample to a stepped intensity pattern. Clearly the density of non-motile cells is no longer constant throughout the sample but shows a marked step between the two regions. The majority of these additional non-motile cells is introduced almost instantaneously as soon as the pattern is applied, as $(\beta_0(\mathcal{I}_{\text{low}}) - \beta_0(\mathcal{I}_{\text{high}}))\rho$ cells can no longer swim within the low light intensity region. Over time more non-motile cells accumulate near the boundary as a fraction of the cells swimming from the high intensity to the low intensity region also turn non-motile.

In order to minimise the complications introduced by intensity dependent non-motile fractions we avoided experiments using very low light intensities, which limited us to speed ratios $v_{\text{slow}}/v_{\text{fast}} > 0.6$.

Supplementary Note 4 Run-and-tumble strain AD4

All of the experimental data presented in the main manuscript used smooth swimming mutant of *E. coli*, i.e. AD10 or DM1. However, the original prediction was actually made for particles performing run-and-tumble motion in 1D [4], so using a wild-type (WT) strain might appear the more natural choice. We therefore also performed experiments with such a WT light controlled strain (AD4), but found that this leads to much more complex results. Supplementary Figure 10 shows speed and density maps for a sample of AD4 illuminated for 17 min with a simple step light pattern. The panel showing the resulting $\rho^s v$ for this pattern immediately highlights that this is not constant in this case.

This becomes even more evident when looking at the corresponding x-profiles in Supplementary Figure 11.

The total density profile displays an almost opposite trend from what would be expected, but highlights a region of strongly increased density in the first tile column on the low intensity side of the boundary. The q-dependence of the swimming speed in this column suggests [5] that the swimmers tumble much more than in uniform light conditions. Lowering the light intensity on a uniformly illuminated also lead to a similarly pronounced q-dependence, whereas an increase in intensity had no obvious effect (other than increasing the speed). This suggests that the bacteria react differently depending on the 'direction' of the intensity change: Lowering the light seems to induce more tumbling, whereas increasing the light does not change the tumbling rate. We speculate that this behavior could be due to 'energy taxis' [6], i.e. bacteria actively adjusting their tumbling rate when they sense that the energy sources are dropping. The prediction of $\rho(x)v(x) = \text{const}$ is based on the assumption that the tumbling rate $\alpha(x)$ is only a function of the position but not the direction (same assumptions are also made about $v(x)$). It is therefore not surprising that we do not find it for this WT strain.

Supplementary References

[1] Arlt, J., Martinez, V. A., Dawson, A., Pilizota, T. & Poon, W. C. K. Painting with light–powered bacteria. *Nat Commun.* **9**, 768 (2018).

[2] Miño, G. *et al.* Enhanced Diffusion due to Active Swimmers at a Solid Surface. *Phys. Rev. Lett.* **106**, 1–4 (2011).

[3] Jepson, A., Martinez, V. A., Schwarz-Linek, J., Morozov, A. & Poon, W. C. K. Enhanced diffusion of nonswimmers in a three-dimensional bath of motile bacteria. *Phys. Rev. E* **88**, 041002(R) (2013).

[4] Schnitzer, M. J. Theory of continuum random walks and application to chemotaxis. *Phys. Rev. E* **48**, 2553–2568 (1993).

[5] Martinez, V. A. *et al.* Differential dynamic microscopy: a high-throughput method for characterizing the motility of microorganisms. *Biophys. J.* **103**, 1637–1647 (2012).

[6] Schweinitzer, T. & Josenhans, C. Bacterial energy taxis: a global strategy? *Arch. Microbiol.* **192**, 507–520 (2010).
